# Reduced survival of young patients under 55 years with metastatic prostate cancer: a population-based study

**DOI:** 10.7150/jca.58144

**Published:** 2021-06-11

**Authors:** Pan Song, Zhufeng Peng, Mengxuan Shu, Jiaxiang Wang, Peiwen Liu, Xiaoyu Di, Luchen Yang, Zhenghuan Liu, Jing Zhou, Qiang Dong

**Affiliations:** 1 Department of Urology, West China Hospital of Sichuan University, Chengdu, 610000, Sichuan Province, China.; 2 The first Clinical Medical College of Lanzhou University, Lanzhou, 730000, Gansu Province, China.; 3 The second Clinical Medical College of Lanzhou University, Lanzhou, 730000, Gansu Province, China.

**Keywords:** metastatic prostate cancer, young patients, prognosis, SEER

## Abstract

**Objective:** The aim of this study was to evaluate the prognosis of patients with metastatic prostate cancer (mPCa) in different age groups.

**Methods:** Patients with mPCa from 2004 to 2016 in the Surveillance, Epidemiology and End Results (SEER) database were identified. Seven groups were divided according to the age at diagnosis, including ≤55 years, 56-60 years, 61-65 years, 66-70 years, 71-75 years, 76-80 years and >80 years. Fine and Gray's competing risks model and Kaplan-Meier analysis were conducted to evaluate the cancer-specific survival (CSS).

**Results:** A total of 36231 patients with mPCa were included. The CSS curves of the overall cohort showed that patients aged **≤**55 years had significantly worse CSS than patients in age groups of 56-60 [HR:0.93 (0.87~1.00), p=0.039], 61-65 [HR:0.91 (0.85~0.97), p=0.003] and 66-70 [HR:0.90 (0.84~0.96), p=0.001]. After removing patients dead for other reasons, the differences of CSS curves between ≤55 years group and 56-70 years groups were not significant. However, the mean survival time of ≤55 years group (55.78±2.48 months) was still shorter than 56-60 years (57.28±2.35 months), 61-65 years (57.64±2.07 months), and 66-70 years (57.11±2.11 months). When stratified by M stages, similar results were found in M1a, M1b and M1c stage groups. According to Fine-Gray competing risks models, patient ≤55 years featured significantly higher sub-distribution hazard ratio (sdHR) than 61-65 years group [sdHR: 0.94(0.88~1.00); p=0.046].

**Conclusions:** The mPCa patients ≤55 years seemed to be associated with worse prognosis in comparison with patients aging 56-70 years.

## Introduction

Prostate cancer (PCa) is the most common malignant tumor in the male genitourinary system, seriously threatening the life and health of men 1, 2. By 2020, it was estimated that more than 191,930 men would be newly diagnosed with PCa and 33,330 cases would be dead for PCa 3. Although there are some radical treatments with excellent long-time prognosis for localized prostate cancer, the prognosis of men with metastatic prostate cancer (mPCa) were rather poor. The common metastatic sites of prostate cancer include bones, distant lymph nodes, liver, and thorax lumbar spine 4, 5. Metastasis will occur in approximately 15-33% initial PCa diagnosis within 2 years and has a significant influence on mortality among the population 6. Androgen deprivation therapy (ADT) is the first-line treatment for men with metastatic prostate cancer, which is usually associated with a good effect at the beginning of treatment 7, 8. The majority of patients with mPCa will progress to castration-resistant prostate cancer (CRPC) despite intensive ADT. Once this disease progresses to the castration-resistant stage, the patients are under a substantially great risk of mortality with a median survival time of 15 months 9.

Although the overall prognosis of mPCa patients is rather poor, the survival time of each individual is quite different. The detailed survival time of patients is affected by many factors, such as age, ethnicity, genetic factors etc. 10, 11. In addition to tumor factors, age is one of the most important factors that has great effects on the prognosis of men with mPCa 10. The incidence of prostate cancer is highly correlated with old age. PCa is very rare among young patients and might be more aggressive in terms of biological behavior. Zheng et al. 11 reported that younger patients were associated with significantly worse outcomes in high-risk group patients. For young patients with mPCa, the detailed survival outcomes remain inconclusive.

In this study, we aimed to analyze the prognosis of mPCa patients in different age groups, and to compare the survival outcomes of young patients with patients of other ages.

## Materials and methods

### Data source

The data of this study were extracted from the SEER database from January 1, 2004, to December 31, 2016. Metastatic PCa patients were retrospectively identified with the software of SEER* STAT. The general information and tumor information were collected.

### Inclusion and exclusion criteria

Inclusion criteria: (1) patients were diagnosed with metastatic PCa (M1 stage). (2) The age of patient at diagnosis was clearly known.

Exclusion criteria: (1) Multiple primary cancers; (2) Important information such as age at diagnosis, M stage, and follow-up time was unknown; (3) The survival status and causes of deaths were unclear at the end of follow up.

### Variables and outcomes

According to the age of patients, we divided the included patients into seven groups: **≤**55 years, 56-60 years, 61-65 years, 66-70 years, 71-75 years, 76-80 years and >80 years.

The main outcome in this study was defined as cancer-specific survival (CSS). The survival time was from the patient's first diagnosis to the patient's death or the last follow-up time (December 31, 2016).

### Statistical analyses

Baseline characteristics in seven age groups were presented with frequency and percentage or median and interquartile range (IQR). The Kaplan-Meier (K-M) analysis was conducted to construct the survival curves, and to calculate survival time and survival rates. Hazard ratios (HRs) and their 95% CIs were used to calculate the degree of risk between different age groups. In order to eliminate the interference of death caused by other reasons except prostate cancer-specific death, we re-analyzed the survival outcomes of the cohort without patients dead for other reasons. The competing risks model with Fine-Gray analysis was also introduced into our analysis. The Fine and Gray's competing risks model provides a useful alternative to Cox regression in the presence of one or more competing risks. It focuses on the cumulative incidence function, which indicates the probability of the event of interest happening before a given time. Subdistribution hazard ratio (sdHR) was calculated with their 95%CI in this model. All statistical analyses in this study were performed with the software of SPSS version 25, Graphprism 7.2 and STATA 14.0. P < 0.05 was considered statistically significant.

## Results

### Patient characteristics

A total of 36231 patients were included. 2712, 3414, 4839, 5118, 5168, 5154 and 9826 patients were in age group of ≤55 years, 56-60 years, 61-65 years, 66-70 years, 71-75 years, 76-80 years and >80 years, respectively. The baseline characteristics of seven groups were summarized in **Table 1**.

### Survival analysis

#### Survival curves and survival time of the overall cohort

**Figure 1:**
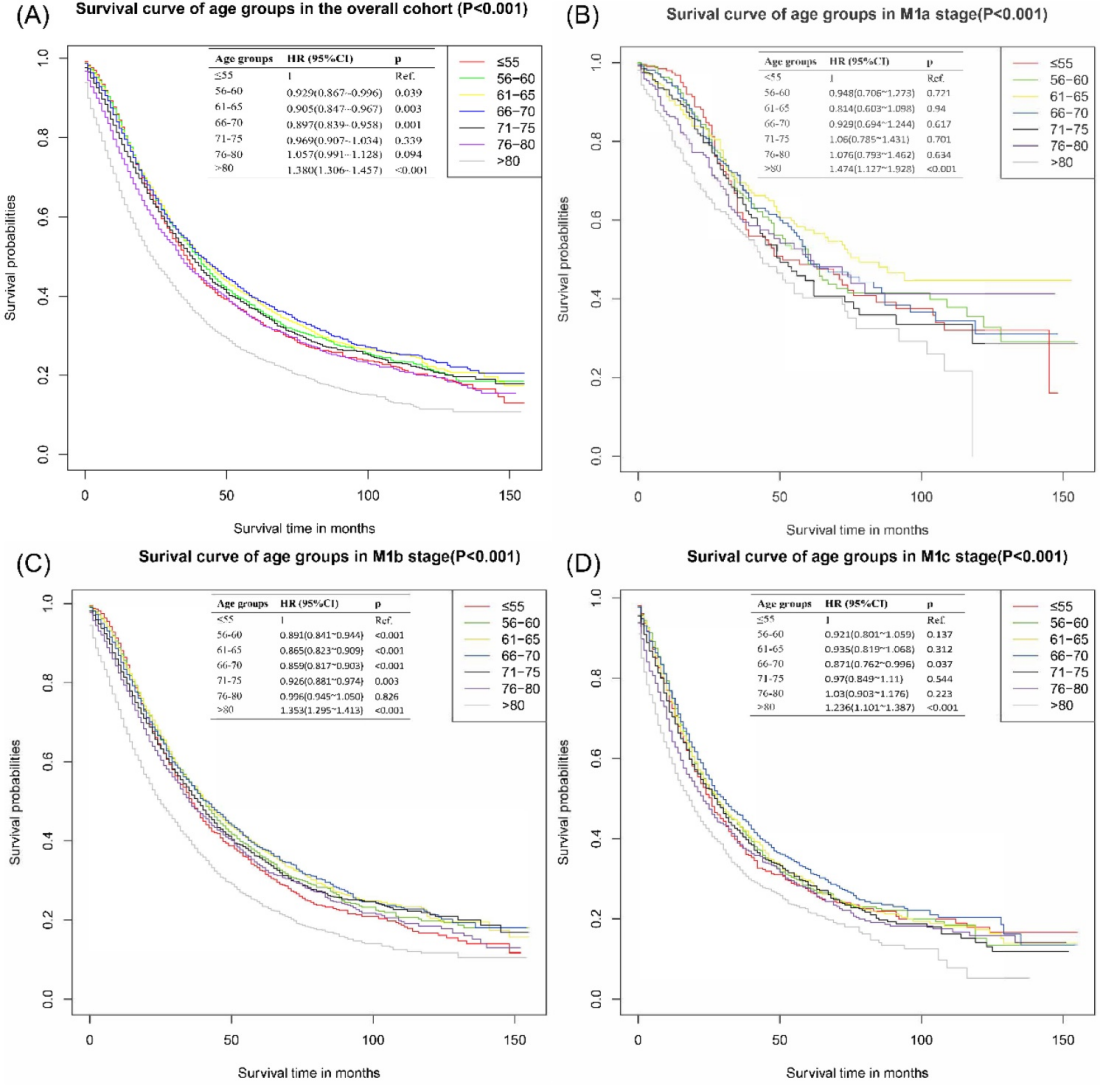
Cancer-specific survival curves of different age groups for patients with metastatic prostate cancer in overall cohort. **(A)** Cancer-specific survival curves of different age groups for patients with metastatic prostate cancer of all M stages. **(B)** Cancer-specific survival curves of different age groups for patients with metastatic prostate cancer of M1a stage in overall cohort. **(C)** Cancer-specific survival curves of different age groups for patients with metastatic prostate cancer of M1b stage in overall cohort. **(D)** Cancer-specific survival curves of different age groups for patients with metastatic prostate cancer of M1c stage in overall cohort.

The survival curves of the overall cohort were presented in **Figure 1A**, patients in **≤**55 years group had significantly worse CSS outcomes than those in 56-60 years group [HR: 0.929, 95% CI (0.867~0.996), p=0.039], 61-65 years group [HR: 0.905, 95% CI (0.847~0.967), p=0.003] and 66-70 years group [HR: 0.897, 95% CI (0.839~0.958), p=0.001]. There was no significant difference between **≤**55 years group and 71-75 years group [HR: 0.969, 95% CI (0.907~1.034), p=0.339] and 76-80 years group [HR: 1.057, 95% CI (0.991~1.128), p=0.094]. The group of age >80 years reported the worst survival outcomes.

Considering the difference of metastatic extensions in the included patients, subgroup analyses based on different M stages were performed. In M1b, significant statistical differences were detected in the comparison of **≤**55 vs 56-60 years group [HR: 0.90, 95% CI (0.84~0.94), p<0.001], **≤**55 vs 61-65 [HR: 0.87, 95% CI (0.82~0.91), p<0.001], **≤**55 vs 66-70 [HR: 0.86, 95% CI (0.82~0.90), p<0.001] and **≤**55 vs 71-75 [HR: 0.93, 95% CI (0.88~0.97), p=0.003], individually. 66-70 years group in M1c also presented an apparently better survival outcome than **≤**55 group with an HR of 0.87 [95% CI (0.76~1.00), p=0.037]. No significantly differences were found in the subgroup of M1a group. Detailed results were shown in **Figure 1B-D**.

The mean survival time of **≤**55 years group was 58.18±2.52 months, significantly shorter than 56-60 years group (61.55±2.41 months), 61-65 years group (62.99±2.14 months), 66-70 years group (64.01±2.2 months) and 71-75 years group (59.87±2.18 months) with all M stages. Similar results were found in the subdivided M stage groups. All of the detailed results were presented in **Table 2**. As is also shown in Table 2, patients **≤**55 years had better 1-year survival rate than other age groups. However, The 2-, 3- and 5-year survival rates in **≤**55 years groups were significantly worse than those in the age group of 56-60 years, 61-65 years, 66-70 years and 71-75 years.

#### Survival curve and survival time of the cohort without patients dead for other reasons

**Figure 2:**
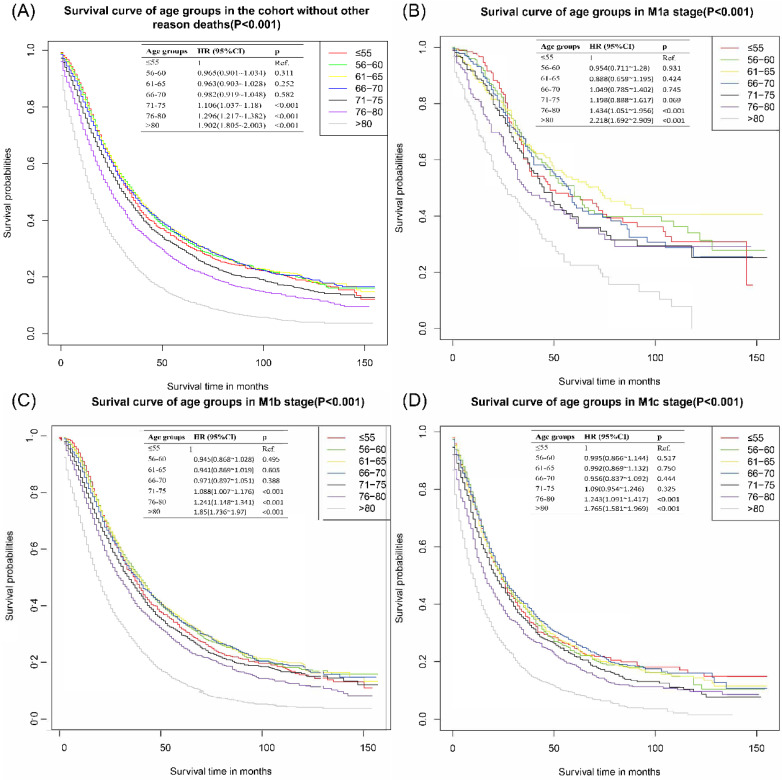
Cancer-specific survival curves of different age groups for patients with metastatic prostate cancer in the cohort that excluded other reason deaths. **(A)** Cancer-specific survival curves of different age groups for patients with metastatic prostate cancer of all M stages without other-reason death. **(B)** Cancer-specific survival curves of different age groups for patients with metastatic prostate cancer of M1a without other-reason deaths. **(C)** Cancer-specific survival curves of different age groups for patients with metastatic prostate cancer of M1b without other-reason deaths. **(D)** Cancer-specific survival curves of different age groups for patients with metastatic prostate cancer of M1c without other-reason deaths.

The survival curve of the cohort without patients dead for other reasons was presented in **Figure 2A**. There were no significant differences among patients ≤55 years, 56-60 years, 61-65 years, and 66-70 years. Patients above 70 years old had worse CSS outcomes than those **≤**55 years old. With **≤**55 years group as the reference, the HR and 95%CI of patients aged 56-60 years, 61-65 years, 66-70 years, 71-75 years, 76-80 years and >80 years were 0.97 (0.90~l.03), 0.96 (0.90~1.03), 0.98 (0.92~1.05), 1.11 (1.04~l.18), 1.30 (1.22~1.38) and 1.90 (1.81~2.00), respectively. In the subgroup analyses of different M stages, there were no significant differences among **≤**55 years, 56-60 years, 61-65 years and 66-70 years groups. These results were presented in **Figure 2B-D**.

The mean survival time of patients **≤**55 years was 55.78±2.48 months, which was slightly shorter than those in 56-60 years group (57.28±2.35 month), 61-65 years group (57.64±2.07 month), and 66-70 years group (57.11±2.11 month). The mean survival time of patients aging 76-80 years and >80 years were 43.79±1.89 month and 27.56±1.1 month, individually. Similar results of survival time were found in M stage subgroups. The survival rate of the cohort without patients dead for other reasons revealed that patients **≤**55 years had slightly better 1-year CSS, similar 2-year CSS, and worse 3- and 5- year CSS than patients in 56-60 years group, 61-65 years group, and 66-70 years group. The survival rate of patients **≤**55 years was better than those aging 71-75 years, 76-80 years and >80 years. All of the detailed results can be accessed to in **Table 2.**

#### Multivariate Fine-Gray analysis

The results of multivariate Fine-Gray analysis in competitive risk model were shown in **Table 3**. The factors, including age, year of diagnosis, race, marital status, PSA levels and Gleason score, were independent prognostic risk factors. With age ≤55 years as the reference, the sdHR and 95%CI of patients aged 56-60 years, 61-65 years, 66-70 years, 71-75 years, 76-80 years and >80 years were 0.95 (0.89~1.02), 0.94 (0.88~0.99), 0.95 (0.9~1.01), 1.04 (0.97~1.11), 1.07 (1.00~1.14) and 1.34 (1.27~1.43), respectively.

## Discussion

Although early-stage PCa can usually be cured by radical surgery, the prognosis for patients with distant metastasis is rather poor even with the optimal treatment. Once prostate cancer reaches the metastatic stage, the average life expectancy of patients will be severely reduced 12. Age is an important factor which has great effects on the prognosis of individuals. mPCa in young patients seems to be more aggressive and poorly differentiated. The prognosis of young patients with mPCa has not been evaluated in detail.

Our results found that the survival outcomes of patients ≤55 years were significantly worse than those of patients aged 56-60 years, 61-65 years and 66-70 years, similar to those aging 71-75 years. These results had been proven by some previous studies. Kimura et al.^13^ found the 5-years overall survival rates in the young group (aged ≤55 years) were significantly worse than those in the middle-aged (aged ≥56 and ≤65 years) and elderly groups (aged ≥66 and ≤75 years) in patients with stage IV disease with metastasis. Hamstra et al. 14 revealed that older age was associated with decreased metastasis and prostate cancer-specific death for men with localized advanced prostate cancer. They found that the cancer in older men was less aggressive, and was independent of other clinical features. Humphreys et al. 15 also showed a trend towards worse survival in patients aged <55 years. On the contrary, some studies found older patients (>70 years or even older) had a poorer prognosis than the younger 16, 17. Guo et al. 18 demonstrated that younger patients (≤70 years old) with bone and lung metastases presented better survival outcomes than those with other types of metastasis or older age.

To avoid the interference caused by other-reason deaths, we reanalyzed the survival outcomes of the cohort without men dead for other reasons and introduced the competitive risk model. Our results reported that young patients (**≤**55 years) still had slightly worse survival outcomes than patients aged 56-60 years, 61-65 years and 66-70 years, especially for patients in 61-65 years group. Similarly, Bernard et al. 19 revealed that men aged <55 years presented second worst prognosis with lower median PCSS and a higher cumulative incidence of death due to PCa compared with patients aged 55-64 years and 65-74 years.

Some previous studies accounted for the results in this study. Compared with older mPCa patients, younger patients were associated with more aggressive and much advanced stages, resulting in worse outcomes 20-22. Though some studies reported more favorable outcomes in younger patients, the differences of detection and screening methods and patient selection may contribute to these discrepancies 23. Another possible explanation for worse prognosis in the men aged ≤55 years in comparison with the older is that the incidence of BRCA1 and BRCA2 mutation is much higher in young men (aged <65 years) 24 and the BRCA1/2 mutation is correlated with poor prognosis. Men with BRCA1/2 mutation were more likely to have a higher Gleason score and worse prognoses than non-BRCA2 carriers.

There were some limitations in our study. First, this was a retrospective analysis along with some unavoidable confounders and risk biases, which may lead to incompletion of clinical information. Second, limited by the data we could obtained, some factors such as detailed treatments, financial situation, etc. were not analyzed in our study. Our results might be influenced by these factors. Therefore, more high-quality studies are needed in the future to verify our results.

## Conclusions

For mPCa, the prognoses of young patients (**≤**55 years) were slightly worse than those aged between 56 and70 years. However, considering the limitation in our study, more high-quality studies are needed in the future.

## Figures and Tables

**Table 1:** Baseline characteristics of included patients in overall cohort

Characteristic	Total	≤55 years	56-60 years	61-65 years	66-70 years	71-75 years	76-80 years	>80 yeras	p
N	36231	2712	3414	4839	5118	5168	5154	9826	
**Year, n (%)**									
2004-2008	11688 (32.3)	894 (33)	1111 (32.5)	1365 (28.2)	1511 (29.5)	1653(32)	1823 (35.4)	3331 (33.9)	<0.001
2009-2012	10639 (29.4)	866 (31.9)	980 (28.7)	1544 (31.9)	1519 (29.7)	1455(28.2)	1465 (28.4)	2810 (28.6)	
2013-2016	13904 (38.4)	952 (35.1)	1323 (38.8)	1930 (39.9)	2088 (40.8)	2060(39.9)	1866 (36.2)	3685 (37.5)	
**Race, n (%)**									
White	27377 (75.6)	1818 (67)	2307 (67.6)	3335 (68.9)	3722 (72.7)	3890(75.3)	4073 (79)	8232 (83.8)	<0.001
Black	6359 (17.6)	758 (27.9)	905 (26.5)	1175 (24.3)	1017 (19.9)	870(16.8)	689 (13.4)	945 (9.6)	
Others	2495 (6.9)	136 (5)	202 (5.9)	329 (6.8)	379 (7.4)	408(7.9)	392 (7.6)	649 (6.6)	
**Marriage, n (%)**									
Married	20103 (55.5)	1307 (48.2)	1652 (48.4)	2524 (52.2)	2958 (57.8)	3064(59.3)	3099 (60.1)	5499 (56)	<0.001
Unmarried	5822 (16.1)	833 (30.7)	935 (27.4)	1083 (22.4)	846 (16.5)	736(14.2)	602 (11.7)	787 (8)	
Separated	7826 (21.6)	385 (14.2)	590 (17.3)	856 (17.7)	944 (18.4)	972(18.8)	1112 (21.6)	2967 (30.2)	
Unclear	2480 (6.8)	187 (6.9)	237 (6.9)	376 (7.8)	370 (7.2)	396(7.7)	341 (6.6)	573 (5.8)	
**Grade, n (%)**									
Well differentiation	165 (0.5)	16 (0.6)	14 (0.4)	31 (0.6)	25 (0.5)	29(0.6)	28 (0.5)	22 (0.2)	<0.001
Moderate differentiation	1856 (5.1)	154 (5.7)	197 (5.8)	282 (5.8)	333 (6.5)	301(5.8)	258 (5)	331 (3.4)	
Poor differentiation	20539 (56.7)	1797 (66.3)	2272 (66.5)	3181 (65.7)	3234 (63.2)	3202(62)	2862 (55.5)	3991 (40.6)	<0.001
Undifferentiated	302 (0.8)	29 (1.1)	30 (0.9)	37 (0.8)	45 (0.9)	33(0.6)	52 (1)	76 (0.8)	
Unclear	13369 (36.9)	716 (26.4)	901 (26.4)	1308 (27)	1481 (28.9)	1603(31)	1954 (37.9)	5406 (55)	
**T stage, n (%)**									
T1-2	17347 (47.9)	1358 (50.1)	1701 (49.8)	2511 (51.9)	2637 (51.5)	2653(51.3)	2536 (49.2)	3951 (40.2)	<0.001
T3-4	7300 (20.1)	687 (25.3)	844 (24.7)	1096 (22.6)	1119 (21.9)	1032(20)	944 (18.3)	1578 (16.1)	
Unclear	11584 (32)	667 (24.6)	869 (25.5)	1232 (25.5)	1362 (26.6)	1483(28.7)	1674 (32.5)	4297 (43.7)	
**N, n (%)**									
N0	17349 (47.9)	1109 (40.9)	1525 (44.7)	2290 (47.3)	2509 (49)	2633(50.9)	2648 (51.4)	4635 (47.2)	<0.001
N1	8307 (22.9)	963 (35.5)	1068 (31.3)	1362 (28.1)	1347 (26.3)	1124(21.7)	948 (18.4)	1495 (15.2)	
Unclear	10575 (29.2)	640 (23.6)	821 (24)	1187 (24.5)	1262 (24.7)	1411(27.3)	1558 (30.2)	3696 (37.6)	
**M, n (%)**									
M1a	1932 (5.3)	194 (7.2)	243 (7.1)	292 (6)	308 (6)	280(5.4)	250 (4.9)	365 (3.7)	<0.001
M1b	24835 (68.5)	1839 (67.8)	2260 (66.2)	3306 (68.3)	3459 (67.6)	3622(70.1)	3536 (68.6)	6813 (69.3)	
M1c	7664 (21.2)	583 (21.5)	778 (22.8)	1019 (21.1)	1107 (21.6)	993(19.2)	1091 (21.2)	2093 (21.3)	
M1,NOS	1800 (5)	96 (3.5)	133 (3.9)	222 (4.6)	244 (4.8)	273(5.3)	277 (5.4)	555 (5.6)	
**PSA, n (%)**									
<20.0 ng/ml	5919 (16.3)	362 (13.3)	529 (15.5)	779 (16.1)	1012 (19.8)	1068(20.7)	943 (18.3)	1226 (12.5)	<0.001
20.0-97.9 ng/ml	8287 (22.9)	569 (21)	759 (22.2)	1121 (23.2)	1183 (23.1)	1196(23.1)	1246 (24.2)	2213 (22.5)	
>97.9 ng/ml	17158 (47.4)	1518 (56)	1787 (52.3)	2399 (49.6)	2338 (45.7)	2217(42.9)	2260 (43.8)	4639 (47.2)	
Unclear	4867 (13.4)	263 (9.7)	339 (9.9)	540 (11.2)	585 (11.4)	687(13.3)	705 (13.7)	1748 (17.8)	
**Gleason score, n (%)**									
≤7	4728 (13)	430 (15.9)	528 (15.5)	719 (14.9)	782 (15.3)	771(14.9)	661 (12.8)	837 (8.5)	<0.001
8-10	17327 (47.8)	1506 (55.5)	1906 (55.8)	2713 (56.1)	2741 (53.6)	2683(51.9)	2414 (46.8)	3364 (34.2)	
Unclear	14176 (39.1)	776 (28.6)	980 (28.7)	1407 (29.1)	1595 (31.2)	1714(33.2)	2079 (40.3)	5625 (57.2)	
**Bone metastasis**									
Yes	19378 (53.5)	1405 (51.8)	1817 (53.2)	2739 (56.6)	2871 (56.1)	2804(54.3)	2589 (50.2)	5153 (52.4)	<0.001
No/Unclear	16853 (46.5)	1307 (48.2)	1597 (46.8)	2100 (43.4)	2247 (43.9)	2364(45.7)	2565 (49.8)	4673 (47.6)	
**Lung metastasis**									0.596
Yes	1784 (4.9)	129 (4.8)	174 (5.1)	265 (5.5)	253 (4.9)	250(4.8)	241 (4.7)	472 (4.8)	
No/Unclear	34447 (95.1)	2583 (95.2)	3240 (94.9)	4574 (94.5)	4865 (95.1)	4918(95.2)	4913 (95.3)	9354 (95.2)	
**Liver metastasis**									0.006
Yes	1007 (2.8)	76 (2.8)	104 (3)	168 (3.5)	150 (2.9)	147(2.8)	132 (2.6)	230 (2.3)	
No/Unclear	35224 (97.2)	2636 (97.2)	3310 (97)	4671 (96.5)	4968 (97.1)	5021(97.2)	5022 (97.4)	9596 (97.7)	
**Brain metastasis**									
Yes	255 (0.7)	25 (0.9)	29 (0.8)	46 (1)	34 (0.7)	33(0.6)	36 (0.7)	52 (0.5)	0.066
No/Unclear	35976 (99.3)	2687 (99.1)	3385 (99.2)	4793 (99)	5084 (99.3)	5135(99.4)	5118 (99.3)	9774 (99.5)	
**Living status**									
Alive	11544 (31.9)	1030 (38)	1323 (38.8)	1937 (40)	2049 (40)	1818(35.2)	1495 (29)	1892 (19.3)	0.012
Cancer-specific death	17774 (49.1)	1507 (55.6)	1756 (51.4)	2331 (48.2)	2333 (45.6)	2413(46.7)	2460 (47.7)	4974 (50.6)	
Other reason death	6913 (19.1)	175 (6.5)	335 (9.8)	571 (11.8)	736 (14.4)	937(18.1)	1199 (23.3)	2960 (30.1)	

PSA: prostate-specific antigen.

**Table 2:** The cancer-specific survival rates and survival time of patients in different age groups

CSS	≤55 years	56-60 years	61-65 years	66-70 years	71-75 years	76-80 years	>80 years
**Survival time of patients in overall cohort (Mean±SD), months**							
All patients	58.18±2.52	61.55±2.41	62.99±2.14	64.01±2.20	59.87±2.18	55.82±2.15	43.38±1.70
M1a stage	76.52±5.00	80.51±4.80	89.99±4.71	79.42±4.49	76.58±5.47	80.03±5.46	56.63±3.4
M1b stage	58.28±1.50	62.19±1.51	63.85±1.31	65.16±1.36	61.56±1.34	57.22±1.32	44.4±1.02
M1c stage	50.80±2.68	53.14±2.46	52.34±2.20	55.57±2.25	48.61±2.18	47.33±2.16	35.75±1.52
**Survival rates of overall cohort,%**							
1- year	84.0% (82.6%~85.4%)	83.7% (82.3%~85.1%)	82.6% (81.4%~83.8%)	82.0% (80.8%~83.2%)	79.8% (78.6%~81.0%)	76.0% (74.8%~77.2%)	66.7% (65.7%~67.7%)
2- year	63.0% (61.0%~65.0%)	64.6% (62.8%~66.4%)	65.7% (64.1%~67.3%)	64.9% (63.3%~66.5%)	63.1% (61.5%~64.7%)	59.4% (57.8%~61.0%)	49.1% (47.9%~50.3%)
3-year	47.9% (45.7%~50.1%)	52.0% (50.0%~54.0%)	52.5% (50.9%~54.1%)	53.3% (51.7%~54.9%)	50.9% (49.3%~52.5%)	47.4% (45.6%~49.2%)	38.2% (37.0%~39.4%)
5- year	32.7% (30.5%~34.9%)	35.4% (33.2%~37.6%)	37.8% (36.0%~39.6%)	38.4% (36.6%~40.2%)	36.2% (34.4%~38.0%)	34.0% (32.2%~35.8%)	24.7% (23.3%~26.1%)
**Survival time of the cohort without patients dead for other reasons (Mean±SD), months**							
All patients	55.78±2.48	57.28±2.35	57.64±2.07	57.11±2.11	50.92±2.03	43.79±1.89	27.56±1.10
M1a stage	74.75±5.01	78.4±4.81	84.45±4.84	71.94±4.53	68.91±5.40	63.73±5.65	39.23±3.06
M1b stage	53.28±1.47	56.50±1.48	56.81±1.29	55.66±1.31	50.70±1.25	44.47±1.16	28.26±0.67
M1c stage	46.57±2.63	44.29±2.28	45.15±2.07	46.55±2.11	39.01±1.91	34.69±1.82	20.23±0.88
**Survival rates of the cohort without patients dead for other reasons, %**							
1- year	83.0% (81.4%~84.6%)	82.1% (80.7%~83.5%)	80.9% (79.7%~82.1%)	80.5% (79.3%~81.7%)	76.0% (74.6%~77.4%)	69.9% (68.3%~71.5%)	55.7% (54.5%~56.9%)
2- year	61.1% (59.1%~63.1%)	61.8% (59.8%~63.8%)	62.9% (61.3%~64.5%)	62.2% (60.6%~63.8%)	57.2% (55.6%~58.8%)	50.6% (48.8%~52.4%)	35.1% (33.9%~36.3%)
3-year	45.7% (43.5%~47.9%)	48.6% (46.6%~50.6%)	49.4% (47.6%~51.2%)	48.2% (46.4%~50.0%)	44.2% (42.4%~46.0%)	37.7% (35.9%~39.5%)	24.1% (22.9%~25.3%)
5- year	30.7% (28.5%~32.9%)	31.9% (29.9%~33.9%)	34.7% (32.9%~36.5%)	32.9% (31.1%~34.7%)	29.4% (27.6%~31.2%)	24.3% (22.5%~26.1%)	12.1% (11.1%~13.1%)

CSS: cancer-specific survival; SD: standard deviation; IQR: interquartile range.

**Table 3:** Multivariate Fine-Gray for patients with metastatic prostate cancer

Risk factors	Fine-Gray model	
	sdHR (95%CI)	p
**Age**		
≤55 years	1	Ref.
56-60 years	0.95 (0.89~1.02)	0.149
61-65 years	0.94 (0.88~0.99)	0.046
66-70 years	0.95 (0.9~1.01)	0.130
71-75 years	1.04 (0.97~1.11)	0.262
76-80 years	1.07 (1.00~1.14)	0.034
>80 years	1.34 (1.27~1.43)	<0.001
**Year of diagnosis**		
2004-2008	1	Ref.
2009-2012	0.91 (0.88~0.94)	<0.001
2013-2016	0.84 (0.81~0.88)	<0.001
**Race**		
White	1	Ref.
Black	1.03 (0.99~1.07)	0.145
Others	0.79 (0.74~0.84)	<0.001
**Marriage**		
Married	1	Ref.
Unmarried	1.18 (1.13~1.24)	<0.001
Separated	1.18 (1.14~1.23)	<0.001
**M**		
M1a	1	Ref.
M1b	1.63 (1.51~1.76)	<0.001
M1c	1.97 (1.81~2.13)	<0.001
**PSA**		
<20.0 ng/ml	1	Ref.
20.0-97.9 ng/ml	1.18 (1.12~1.25)	<0.001
≥98.0 ng/ml	1.47 (1.4~1.55)	<0.001
**Gleason score**		
≤7	1	Ref.
8-10	1.51 (1.43~1.59)	<0.001

HR: hazard ratio; sdHR: subdistribution hazard ratio; 95% CI: 95% confidence interval; Ref: reference; PSA: prostate-specific antigen.
